# Population Structure and Genomic Breed Composition in an Angus–Brahman Crossbred Cattle Population

**DOI:** 10.3389/fgene.2018.00090

**Published:** 2018-03-27

**Authors:** Mesfin Gobena, Mauricio A. Elzo, Raluca G. Mateescu

**Affiliations:** Department of Animal Sciences, University of Florida, Gainesville, FL, United States

**Keywords:** population structure, genomic breed composition, cattle, Angus, Brahman

## Abstract

Crossbreeding is a common strategy used in tropical and subtropical regions to enhance beef production, and having accurate knowledge of breed composition is essential for the success of a crossbreeding program. Although pedigree records have been traditionally used to obtain the breed composition of crossbred cattle, the accuracy of pedigree-based breed composition can be reduced by inaccurate and/or incomplete records and Mendelian sampling. Breed composition estimation from genomic data has multiple advantages including higher accuracy without being affected by missing, incomplete, or inaccurate records and the ability to be used as independent authentication of breed in breed-labeled beef products. The present study was conducted with 676 Angus–Brahman crossbred cattle with genotype and pedigree information to evaluate the feasibility and accuracy of using genomic data to determine breed composition. We used genomic data in parametric and non-parametric methods to detect population structure due to differences in breed composition while accounting for the confounding effect of close familial relationships. By applying principal component analysis (PCA) and the maximum likelihood method of ADMIXTURE to genomic data, it was possible to successfully characterize population structure resulting from heterogeneous breed ancestry, while accounting for close familial relationships. PCA results offered additional insight into the different hierarchies of genetic variation structuring. The first principal component was strongly correlated with Angus–Brahman proportions, and the second represented variation within animals that have a relatively more extended Brangus lineage—indicating the presence of a distinct pattern of genetic variation in these cattle. Although there was strong agreement between breed proportions estimated from pedigree and genetic information, there were significant discrepancies between these two methods for certain animals. This was most likely due to inaccuracies in the pedigree-based estimation of breed composition, which supported the case for using genomic information to complement and/or replace pedigree information when estimating breed composition. Comparison with a supervised analysis where purebreds are used as the training set suggest that accurate predictions can be achieved even in the absence of purebred population information.

## Introduction

Around 40% of all beef cattle in the United States are located in the subtropical southern and southeastern parts of the country (Cundiff et al., 2012). The combined effect of high ambient temperature and humidity, increased abundance of parasitic and parasite-transmitted diseases, and nutritional lower quality pastures has a negative effect on growth rate and reproductive performance on Taurine (*Bos taurus*) beef cattle breeds of European origin (Burrow, 2015). Crossbreeding between European Taurine and Zebu (*Bos indicus*) breeds is a common strategy used to enhance beef production in tropical and subtropical areas (Lamy et al., 2012). These crossbreds combine the production performance of Taurine cattle with the tropical adaptation of Zebu cattle, and usually outperform purebred cattle from the parental breeds in subtropical conditions due to heterosis (Burrow, 2015). Angus–Brahman crosses are typically better suited for beef production than other Zebu–Taurine combinations in subtropical parts of the United States (Chase et al., 2004). Whether a particular Taurine–Zebu combination is optimal depends on the specific production environment under consideration. It has been suggested (Cundiff et al., 2012) that, in subtropical regions of the United States such as the Gulf Cost, cattle with a 1:1 Taurine to Zebu ratio would be preferred whereas a 3:4 Taurine to Zebu ratio would be better suited to the more northern but still subtropical parts of the United States such as Southeastern Oklahoma and most of Texas.

Having accurate knowledge of breed composition is essential in evaluating the adaptability of crossbreds to a given production environment (Kuehn et al., 2011). Pedigree information is conventionally used to determine breed composition in crossbred cattle (Frkonja et al., 2012; Vanraden and Cooper, 2015). However, the reliability of pedigree-based estimation of breed composition can be compromised by missing, inaccurate, or incomplete records (Vanraden and Cooper, 2015). In addition, Mendelian sampling during gametogenesis can lead to deviations from the breed composition expected from the pedigree (Kuehn et al., 2011).

Using genomic data to determine breed composition is superior to using pedigree records as it was shown to be more accurate whilst not being prone to missing, incomplete, or inaccurate records (Kuehn et al., 2011; Dodds et al., 2014; Funkhouser et al., 2017). Another use of genomic information could be independent authentication of breed in breed-labeled beef products (Wilkinson, 2012). Disadvantages of using genomic data may include genotyping cost and the need for more advanced technical expertise, but both are decreasing in importance as genetic and genomic methods are becoming widely adopted and increasingly accessible (Wiggans et al., 2011). The increasing availability of core sequencing facilities at academic and research institutes, combined with the availability of affordable genotyping services from biotech companies, are likely to improve the accessibility and feasibility of using genomic information to determine breed composition (Gould, 2015; Bauck, 2016). Genotyping cost can also be further reduced by only genotyping breed-informative markers (Wilkinson et al., 2011). Using a small number of carefully selected breed-informative markers is also advantageous in that it minimizes statistical noise coming from other markers whose frequency has been affected by demographic events that are not relevant to breed membership inference (Wilkinson et al., 2011).

The goal of the current study was to determine genomic breed composition while accounting for familial relationships in a group of 782 cattle with substantial presence of such relationships, and in the process, demonstrate the effect of familial relationships on population structure and genomic breed composition inferences. The objectives of the study were to (1) use genomic data to detect population structure due to differences in breed composition by means of parametric and non-parametric methods while accounting for the confounding effect of close familial relationships, and (2) compare breed composition inferred from genomic data with breed composition derived from pedigree.

## Materials and Methods

### Animals and Genotyping

The study included 782 cattle from the multibreed Angus–Brahman herd at the University of Florida (Elzo and Wakeman, 1998). The research protocol was approved by the University of Florida Institutional Animal Care and Use Committee number 201003744. This herd was constructed using a diallel crossbreeding scheme, where six groups of sires with different proportions of Angus and Brahman, as determined from pedigree records, were reciprocally mated with six dam groups which were classified in the same manner as the sires (Komender, 1988; Elzo and Wakeman, 1998). The six sire/dam breed groups were: group 1 (>4/5 Angus); group 2 (3/4 Angus and 1/4 Brahman); group 3 (5/8 Angus and 3/8 Brahman); group 4 (1/2 Angus and 1/2 Brahman); group 5 (1/4 Angus and 3/4 Brahman); and group 6 (>4/5 Brahman). The progeny from the diallel matings were again classified into six groups using the same criteria as the sire/dam groups. The animals included in the current study were sampled to be representative of all six sire/dam/progeny groups and consisted of 126, 120, 123, 159, 84, and 170 cattle from groups 1–6, respectively. Sire group 3 represents Brangus cattle, which is technically a separate breed. However, since Brangus is a composite breed derived from Angus and Brahman, the Brangus cattle and their progeny were considered as crossbreds between the two parental breeds as recommended by Kuehn et al. (2011).

Genomic DNA was extracted from blood samples using the QIAGEN^®^ DNeasy^®^ kit (Qiagen, Valencia, CA, United States), and genotyping was carried out using the GeneSeek Genome Profiler F-250 (GeneSeek, Inc., Lincoln, NE, United States). Several quality control (QC) measures were applied to genotype data at the animal and marker level (Anderson et al., 2010). At the animal level, QC filters included genotype completion rate (<90%) and duplicate removal (pairwise IBS > 0.99). Per-marker filters were applied for minor allele frequency (MAF; <1%), genotype call rate (<90%), and Hardy–Weinberg equilibrium deviation (Chi-square *P*-value <1 × 10^-8^). The extent of linkage disequilibrium (LD) was evaluated by observing LD decay as a function of genomic physical distance. Markers in high LD were then pruned with window size of 5,000 kb, step size of 10 bp, and LD threshold of 0.5 (Turner et al., 2011). All QC steps were performed using the software PLINK1.9 (Chang et al., 2014). The genotype data is available on the EVA website, accession number PRJEB24746.

After removing duplicates (IBS > 0.99; *n* = 2) and samples with low genotype completion rate (<0.9; *n* = 104), 676 samples were available for subsequent analysis. From an initial set of 221,077 SNP, a subset of 89,728 SNP was kept after removing 64,496 SNP with low MAF (<1%); 13,386 SNP for failing to meet minimum call rate (<0.9); 8,088 SNP for Hardy–Weinberg equilibrium deviation and 45,379 SNP due to LD pruning.

### Selection of Unrelated Animals

In both model-based analysis and principal component analysis (PCA), efforts to identify population structure due to differences in breed composition can be biased by the presence of close familial relationships and shared recent ancestry among the sample set being analyzed (Patterson et al., 2006; Conomos et al., 2016). These type of confounding relationships among animals were expected in these data. To account for such sources of confounding in both model-based analysis and PCA, the population structure identified in a subset of unrelated samples was used as a reference when inferring genomic breed composition for the rest of the samples.

A subset of mutually unrelated animals that is representative of overall population structure in the entire sample set was identified using an algorithm described by Conomos et al. (2016) and implemented in the “pcairPartition” function from the R package Genesis. This algorithm utilizes a pairwise kinship matrix estimated by the KING-robust method (Manichaikul et al., 2010) to identify a subset of mutually unrelated samples. Unlike kinship estimation methods which assume a homogeneous population with no structure (e.g., IBD estimation implemented in PLINK; Chang et al., 2014), the KING-robust method is not confounded by the presence of population structure. Moreover, when applied to a set of samples with heterogeneous breed ancestry, the KING-robust method gives a systematically biased negative estimate (termed divergence) for a given pair of unrelated samples with different breeds of origin. The “pcairPartition” algorithm uses this informative bias to include samples with divergent ancestry in the unrelated set in order to represent overall population structure (Conomos et al., 2016). Samples in the unrelated set were selected to have pairwise kinship coefficients of less than 0.022 among them, whilst having the largest number of pairwise divergences of less than -0.022 with the rest of the samples (Conomos et al., 2016). Pairwise kinship and divergence was calculated using the R function “snpgdsIBDKing” in the package Genesis (Conomos et al., 2016).

### Model-Based Analysis

Individual breed composition was estimated from genomic data using a maximum likelihood model implemented in the software ADMIXTURE v1.3 (Alexander et al., 2009; Shringarpure et al., 2016). ADMIXTURE uses genotype data to cluster individuals into subgroups, with the expected number of subgroups (*K*) specified beforehand. Subgroup memberships were considered as breed membership, and pedigree information was used to identify the breed associated with a subgroup. Estimates of genomic breed composition and marker allele frequency were obtained from the *Q* and *F* matrices. The *Q* matrix contains membership coefficients of samples to each subgroup/breed. Since fractional subgroup membership is allowed, membership coefficients can also be conveniently interpreted as the proportion of an animal’s genome with a particular breed ancestry. The *F* matrix contains allele frequency estimates for each marker in each subgroup/breed.

The accuracy of the genomic breed-composition estimation using the maximum likelihood model of ADMIXTURE depends on the extent to which several underlying assumptions are met. These include a relative level of linkage equilibrium between markers, a sample set with adequate representation of all parental/ancestral breeds and a sample set composed of only unrelated animals (Alexander et al., 2009). The sampling scheme employed in the current study ensures adequate representation of both Angus and Brahman, whereas the LD pruning step performed as part of QC is expected to minimize the effect of widespread admixture LD in the sample set (Alexander et al., 2009).

To account for the confounding effect of known and/or cryptic familial relationships, population structure identified in the unrelated subset was used as a reference when inferring genomic breed composition in the related set (Shringarpure et al., 2016). ADMIXTURE was first run in unsupervised mode on the unrelated subset, and breed allele frequencies estimated from this run were then used as inputs for a supervised run on the related set in supervised mode.

To infer breed composition using the projection method, ADMIXTURE was run in the unsupervised mode on the unrelated subset, using genotype data and a *K* = 2 as inputs, and individual breed membership coefficient (*Q*) and breed allele frequency (*F*) estimates were obtained. Genotype data for the remaining samples was then projected onto the population structure inferred for the unrelated samples (Shringarpure et al., 2016). This was accomplished by using the breed allele frequencies (*F*) estimated for the unrelated set, along with genotype data for the rest of the samples and a *K* = 2 as inputs when estimating breed membership coefficients (*Q*) for the rest of the samples. A *K* = 2 was used based on our knowledge of two parental breeds used for all animals in the study (Patterson et al., 2006; Zheng and Weir, 2016).

An additional projection was used where ADMIXTURE was run in the unsupervised mode on a subset of 44 purebred Angus and 44 purebred Brahman animals to obtain breed allele frequency (*F*) estimates. Genotype data for the remaining, non-purebred samples was then projected onto the population structure inferred for the purebred samples.

### Principal Component Analysis

Major axes of variation that explain most of the genetic structure in the study population were identified using PC-AiR, a PCA method implemented in the “pcair” function from the R package Genesis (Conomos et al., 2016). The method minimizes confounding effect of close familial relationships and shared recent ancestry by using the population structure identified in a subset of unrelated samples as a reference for population structure for the rest of the samples. PCA was first performed on the genotype data for the unrelated subset by applying eigendecomposition on the covariance matrix of the standardized genotype data (i.e., each SNP column mean centered and divided by the standard deviation). Principal components (PC) for the rest of the samples (i.e., related subset) were then obtained by projecting their standardized genotype data onto the SNP weight matrix calculated for unrelated set in the previous step (Conomos et al., 2016). Overall structuring of genetic variation was visualized in a scatterplot of the top few PCs.

To evaluate the agreement between different methods of inferring breed composition, the top PC from PC-AiR and the breed composition estimates from the unsupervised ADMIXTURE was compared to the genomic breed composition estimates from the supervised ADMIXTURE (trained on purebred animals) and the pedigree-based breed composition. The agreement between the different methods was evaluated by computing pairwise Pearson’s correlation coefficients.

## Results

### Selection of Unrelated Animals

**FIGURE 1:**
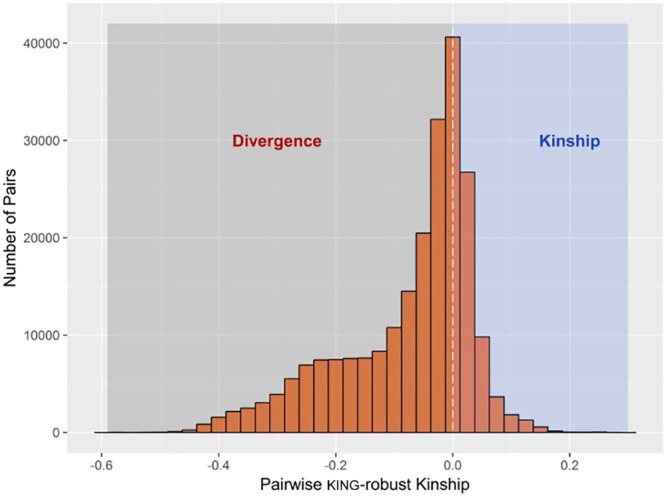
Histogram showing the distribution of pairwise kinship and divergence estimates by KING-robust method among all samples. A total of 74 samples designated as the unrelated set were selected to have pairwise kinship coefficient of less than 0.022 among them, whilst having the largest number of pairwise divergence of less than –0.022 with the rest of the samples.

The R function “pcairPartition” identified a subset of 74 samples as unrelated and representative of the population structure in the entire sample set. The selection was based on pairwise kinship and divergence estimates by the KING-robust method. The distribution of KING-robust estimated for all unique pairwise comparisons among the 676 study animals (*n* = 228,150) is shown in **Figure 1**.

### Model-Based Analysis

**FIGURE 2:**
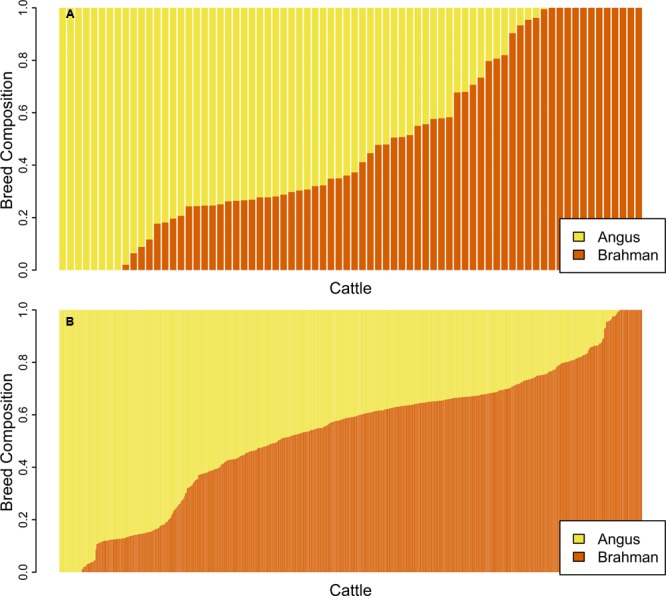
Bar plot of the *Q* matrix from an unsupervised **(A)** ADMIXTURE run showing the proportion of the genome contributed by each breed for 74 unrelated samples. Panel **(B)** shows the *Q* matrix from a supervised run for 602 samples in the related set. Each vertical bar represents an individual with yellow showing the proportion of the genome with Angus ancestry and orange corresponds to the proportion of the genome with Brahman ancestry.

ADMIXTURE was first run in unsupervised mode on the genomic data for the unrelated subset of samples and **Figure 2A** shows the proportion of the genome contributed by each breed for 74 unrelated samples. ADMIXTURE was also run in unsupervised mode on the genomic data for the purebred subset of samples. In the supervised mode, breed allele frequencies estimated for the unrelated or the purebred subset were used as input to estimate breed membership coefficient for the 602 samples in the related set (**Figure 2B**) or the 588 samples in the non-purebred set. As expected (Conomos et al., 2016), the unrelated set of animals identified by pcairPartition was representative of the overall population structure, and contained all animals with membership coefficients of 0 or 1 for both breeds.

**FIGURE 3:**
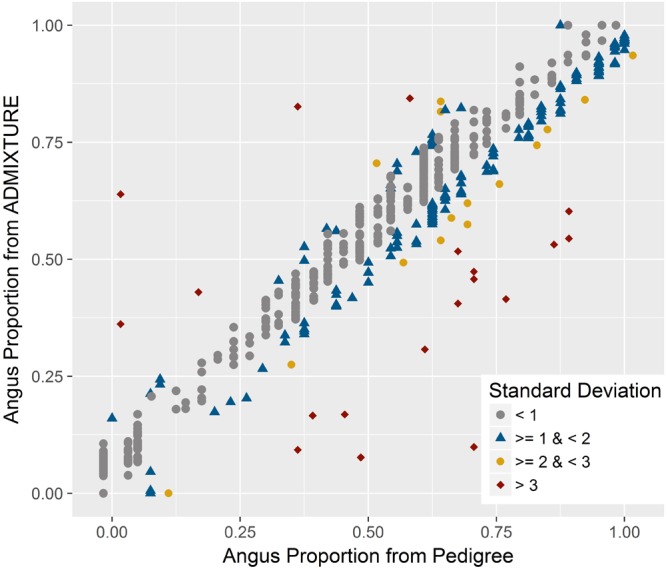
Relationship between genomic breed composition estimates from ADMIXTURE and pedigree data. A very strong positive correlation was found between these estimates with few discrepancies. The color for each animal corresponds to the amount of standard deviation by which the two measures differ.

There was a very strong correlation (*R* = 0.965) between breed composition estimates for either breeds from ADMIXTURE and the estimates obtained using pedigree records. As indicated by the less than perfect correlation, there were discrepancies between the estimates from the two methods for certain samples (**Figure 3**). The mean and standard deviation of the absolute difference between breed composition estimates from the two methods were 0.056 and 0.060, respectively. For 72% of the animals, the difference was within one standard deviation, and 5% had a difference of more than two standard deviations. The correlation between the breed composition estimates from the pure-bred and unrelated trained ADMIXTURE analyses was very strong (*R* = 0.994).

### Principal Component Analysis

**FIGURE 4:**
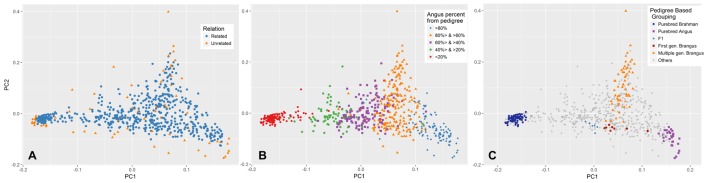
Population distribution across the first (PC1) and second principal component (PC2). **(A)** PCA was performed on the unrelated set of animals (blue) and PC1 and PC2 values for the rest of animals (orange) were predicted based on their genetic similarity to animals in the unrelated set. **(B)** Animals are labeled based on their pedigree breed composition which can be described by the variation in PC1. **(C)** Brangus cattle from one or more generations of Brangus–Brangus matings show more scatter across PC2. In contrast, first generation Brangus and F1 cattle showed minimal scatter across PC2 and were close to the line connecting the two purebred clusters.

The first and second PCs (PC1 and PC2) explained 27 and 5.6% of the variation in the entire genetic data, respectively. Both unrelated and related subsets show a similar pattern of variation across these two PCs (**Figure 4A**). The PC1 had a very strong correlation (*R* = 0.966) with the Angus proportion derived from pedigree data. The relationship between PC1 and pedigree breed composition is also illustrated in **Figure 4B**. Animals with approximately 2/3 Angus ancestry (which would qualify as Brangus) had more variation in PC2, indicating the presence of a source of population structure other than Angus–Brahman differentiation. This variation could be attributed to the distance of Brangus animals from the initial crossbreeding forming a Brangus animal, with first generation Brangus animals being closest to the average, and the distance from the average increasing with the number of generations from this initial Brangus formation (**Figure 4C**). In contrast, F1 and first generation Brangus cattle were located along the line connecting the two clusters formed by the purebreds (**Figure 4C**) and showed much less variation across PC2 as would be expected in the case of a recent two-way admixture (Patterson et al., 2006; McVean, 2009).

**FIGURE 5:**
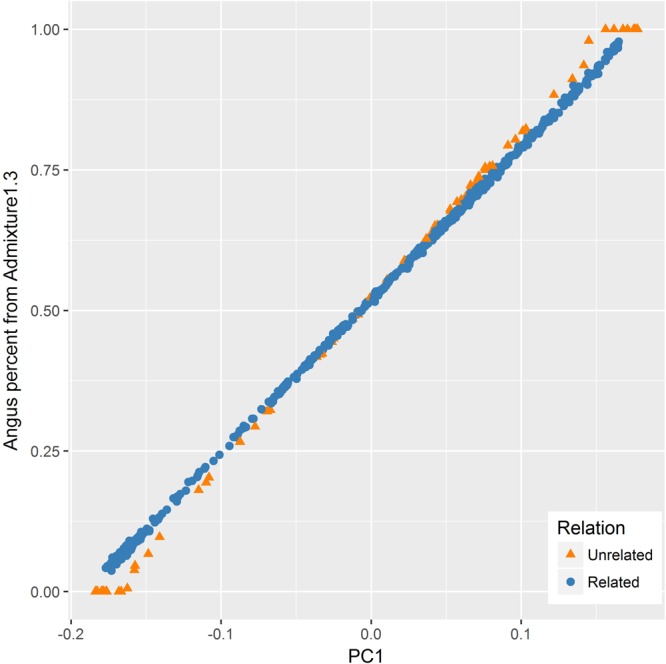
Relationship between the first principal component (PC1) and Angus percent estimated by ADMIXTURE for the related (blue) and unrelated (orange) set of samples. Angus proportion from ADMIXTURE tended to be more extreme as compared to PC1 values for the unrelated set for animals with a higher percentage of one particular breed (purebreds).

The PC1 had also a very strong correlation (*R* = 0.999) with the Angus proportion estimated from ADMIXTURE. However, the relationship between the two values appeared to be different for the related and unrelated subset of samples (**Figure 5**). For the related set, there was a linear relationship between the two values, whereas for the unrelated set, ADMIXTURE estimates had more extreme values at both ends.

## Discussion

A commonly used approach in genomic breed composition inference in livestock is to use regression-based methods which depend on the availability of allele frequency estimates from reference populations. However, there are multiple scenarios where this may not be possible, for example when the full range of ancestral breeds is not known. Even when this information is known, allele frequency estimates from reference populations may not be available for the breeds or genetic markers of interest. One viable option in such cases is to use unsupervised model-based clustering methods which simultaneously estimate breed allele frequency and sample breed membership coefficients (i.e., genomic breed composition). In addition to not requiring allele frequency estimates from external reference populations, such methods allow inference on the correct number of ancestral populations, an important feature when the full range of parental breeds involved is not certain.

Unsupervised model-based clustering used to infer genomic breed composition runs a higher risk of confounding by sources of population structure other that heterogeneous breed ancestry. This is because, unlike the regression methods mentioned earlier, breed allele frequencies are not explicitly specified, and familial relationships among the sample can bias cluster (i.e., breed) allele frequency estimates. Assuming the sample population is large enough, one way to control for such confounding is to base genomic breed composition inference on a subset of mutually unrelated and ancestrally related samples. This involves estimating cluster (i.e., breed) allele frequencies using the unrelated subset in an unsupervised run, and using allele frequency estimates from this run as an input in a supervised run involving the rest of the samples. A useful feature of the KING-robust estimator used in the present study is that it gives out negative estimates (i.e., divergence) for unrelated pairs with different breed ancestry, and this relationship is inversely proportional such that the more divergent the ancestry between the pairs, the more negative the divergence estimate. The extent of both familial relationships and ancestral heterogeneity in the sample is shown in **Figure 1**. Based on these estimates, pcairPartition identified 74 samples as unrelated and ancestrally representative of the entire sample set. This is expected to minimize the confounding effect of close familial relationships on breed membership inference for the related set of samples.

There was a very strong correlation (*R* = 0.965) between breed composition estimates for either breeds from ADMIXTURE and the estimates obtained using pedigree records, which is in agreement with results from other studies. Frkonja et al. (2012) compared different methods of estimating breed composition using purebred Red Holstein Friesian, purebred Simmental, and their crossbreds. This study reported a correlation coefficient of 0.972 between breed proportions obtained from pedigree and breed membership coefficients estimated by STRUCTURE using 40,492 genome-wide SNPs. Another study (Dodds et al., 2014) found a correlation coefficient of 0.89 between breed composition estimates from pedigree and from STRUCTURE run on a set of 10,000 SNP for a set of four different breeds of sheep and their crossbreds.

However, similar to the other studies, there were discrepancies between breed composition estimates from genome-wide data and pedigree for certain samples (**Figure 3**). For crossbred animals, breed composition derived from genomic data should be more accurate than pedigree-based estimates since pedigrees can be incomplete or incorrect (Frkonja et al., 2010; Vanraden and Cooper, 2015). Mendelian sampling during recombination could also lead to deviation from the composition expected based on pedigree (Kuehn et al., 2011). On the other hand, estimates based on genomic data could also be biased or lose accuracy due to sample selection, which could be described as a failure to include sufficient samples to represent all parental breeds in the analysis (Long, 1991; Shringarpure et al., 2016) or a weak differentiation between parental breeds (Patterson et al., 2006; Kuehn et al., 2011). The very high correlation (*R* = 0.994) between breed composition estimates obtained from ADMIXTURE when either an unrelated subset or a set of purebred parental populations was used, suggests that the model-based analyses provide highly accurate estimates of breed composition. In addition, a very high correlation of estimates from both of these methods and estimates from a completely unsupervised ADMIXTURE analysis of the entire dataset (*R* = 0.996 between unsupervised and trained on purebred set, *R* = 0.995 between unsupervised and trained on unrelated subset) suggest the model-based analysis is robust and should be expected to generate highly accurate estimates even in the absence of a training unrelated or purebred dataset.

The PC1 in our PCA explained 27% of the variation in the genetic data, consistent with two major parental breeds (Patterson et al., 2006; McVean, 2009). In a two-way admixture, PC1 is closely related to the *F*st (Wright’s Fixation Index, ratio of between-population variation to overall variation) estimated between the two parental populations. Consistent with McVean’s (2009) observation, the genome-wide *F*st average (0.25) calculated based on the Weir and Cockerham method implemented in PLINK (Weir and Cockerham, 1984) was close to the proportion of variation explained by PC1 (0.27). The PC1 had a strong correlation (*R* = 0.966) with breed composition derived from pedigree, with some discrepancies likely due to inaccuracies in pedigree records and/or the effect of Mendelian sampling (Patterson et al., 2006; Kuehn et al., 2011; Vanraden and Cooper, 2015). An interesting finding related to the ancestry of Brangus (62.5% Angus, 37.5% Brahman) animals is revealed when examining the variation explained by PC2. The highest amount of variation in PC2 is found in Brangus animals and it describes the ancestry of the animals and their distance from first generation Brangus animals, with animals of multiple generations of Brangus–Brangus matings being more distant from first generation Brangus (**Figure 4C**). First generation Brangus cattle showed less variation across PC2, and were located along the line connecting the two clusters formed by the purebreds (**Figure 4C**), as would be expected in the case of a recent two-way admixture (Patterson et al., 2006; McVean, 2009). The distinct pattern of variation seen in the cattle born from Brangus–Brangus mating is likely due to the extended number of generations since the initial crossing of the parental breeds in these animals (Patterson et al., 2006). Close familial relationships can be ruled out as a reason because a similar pattern was also observed in the unrelated set of samples (**Figure 4A**). This result is in agreement with McVean (2009) that, in populations resulting from a two-way admixture, the proportion of genetic variation explained by the PC1 drops as the number of generations since the initial admixture event increases. This could also explain why animals from at least one generation of Brangus–Brangus mating have little variation across PC1 as compared to PC2.

The PC1 had a very strong correlation (*R* = 0.999) with Angus–Brahman proportion from ADMIXTURE, similar to Patterson et al. (2006), reporting a correlation coefficient of 0.995 between PC1 and model estimates for European ancestry in an admixed human population. Despite apparent differences in their approach, both PCA and model-based methods are closely related and can be viewed as different ways of factorizing the genotype matrix (Engelhardt and Stephens, 2010). While the PC1 from PCA is sufficient to measure the level of admixture in a crossbred population with two parental breeds, model-based methods need two coefficients of membership for both breeds to provide the same information (Patterson et al., 2006; Engelhardt and Stephens, 2010).

Notwithstanding the strong correlation between PC1 and breed membership coefficients estimated by ADMIXTURE, the relationship between the two appeared to be different for the related and unrelated set of samples as illustrated in **Figure 5**. For the related set, there was a linear relationship between the two values, whereas for the unrelated set, ADMIXTURE estimates had more extreme values at both ends. A similar observation was made by Engelhardt and Stephens (2010) who noticed that, when applied to an admixed set of samples with divergent ancestral groups, ADMIXTURE tends to give cluster membership estimates that are more extreme as compared to components from PCA. One factor contributing to this tendency is the difference in the type of constraints imposed during optimization when estimating the *Q* matrix and PCs in ADMIXTURE and PCA, respectively.

Because ADMIXTURE needs to explain overall genetic variation or population structure in terms of ancestry from a predefined number of breeds (*K*), ADMIXTURE estimates membership coefficients to all *K* breeds using a constrained optimization process (via quadratic programming) which forces the coefficients to be non-negative and to sum to 1 (Alexander et al., 2009). This means that overall genetic variation is represented only by *K* variables corresponding to membership coefficients for the respective breeds, without attributing variation to any other source. In contrast, PCA does not impose the constraint that a predefined number of PCs have to explain all the genetic variation (Engelhardt and Stephens, 2010). Instead, when applied to data with *n* individuals, PCA estimates *n* PC, each explaining certain proportion of the overall genetic variation. In a two-way admixture, as in this study, PC1 captures genetic variation due to heterogeneous breed ancestry (Patterson et al., 2006). However, variation due to additional factors such as familial relationships are also captured by subsequent PC (McVean, 2009; Engelhardt and Stephens, 2010). Consequently, PC1 values tend to be less biased toward either end of the admixture spectrum as compared to membership coefficient estimates by ADMIXTURE for both ancestral groups (Engelhardt and Stephens, 2010). Another factor contributing to the relatively extreme nature of ADMIXTURE estimates could be its assumption that the errors have a binomial distribution whereas PCA assumes the errors have a Gaussian distribution (Engelhardt and Stephens, 2010).

Compared to ADMIXTURE, PCA is more appealing because it is computationally more efficient while providing a similar level of information as model-based clustering (Patterson et al., 2006). Furthermore, visual representations based on the top few PC provide better insights into the diversity and extent of demographic events underlying different levels of population structure (McVean, 2009). One disadvantage of using PCA is the difficulty in interpreting the results (Patterson et al., 2006). For example, in the current study, cluster membership coefficients estimated by ADMIXTURE were interpreted as Angus and Brahman proportions, but such interpretation cannot be made for PC1 values ranging from -0.18 to 0.18. Although there have been suggestions (Patterson et al., 2006; McVean, 2009; Zheng and Weir, 2016) on how to interpret PCA results in terms of admixture levels or genealogy, caution should be taken when doing so because different demographic events could result in similar PCA projections (McVean, 2009).

## Conclusion

By applying PCA and the maximum-likelihood method of ADMIXTURE to genomic data, it was possible to successfully characterize population structure resulting from heterogeneous breed ancestry, while accounting for close familial relationships. PCA results offered better insight into the different hierarchies of genetic variation structuring. While PC1 was strongly correlated with Angus–Brahman proportions, PC2 represented variation within animals that have a relatively more extended Brangus lineage—indicating the presence of a distinct pattern of genetic variation in these cattle. In contrast, ADMIXTURE estimates of breed composition forced all genetic variation to be explained only in terms of Angus and Brahman proportion, without accounting for other sources of variation. This shows how breed composition inferences made by ADMIXTURE-like methods (e.g., STRUCTURE, fastSTRUCTURE, and Frappe) can be confounded by other sources of population structure and highlights the importance of accounting for such sources by using an unrelated, breed-representative reference population. Although there was strong agreement between breed proportions estimated from pedigree and genetic information, there were significant discrepancies between these two methods for certain animals. This was likely due to inaccuracies in the pedigree-based composition of these animals, which supports the case for using genomic information to complement and/or replace pedigree information when estimating breed composition.

## Author Contributions

MG conducted the analysis and drafted the manuscript. RM conceived and assisted with the analysis and the manuscript. ME assisted with the analysis and the manuscript.

## Conflict of Interest Statement

The authors declare that the research was conducted in the absence of any commercial or financial relationships that could be construed as a potential conflict of interest.
